# Navigating the Complexity of Stiff Person Syndrome: A Case Report and Literature Review

**DOI:** 10.7759/cureus.73219

**Published:** 2024-11-07

**Authors:** Jordyn Mullins, Cody Sheppard, Brian R Beyer, Cheri Blacksten

**Affiliations:** 1 Family Medicine, Burrell College of Osteopathic Medicine, Las Cruces, USA

**Keywords:** gad65, pernicious anaemia, stiff person syndrome, stiff-person syndrome, type i diabetes mellitus

## Abstract

Stiff person spectrum disorder is a disease that involves a host of conditions that are associated with glutamic acid decarboxylase 65-kilodalton isoform autoantibodies. These conditions may include diabetes mellitus type 1, pernicious anemia, autoimmune leukoencephalopathy, cerebellar ataxia, and stiff-person syndrome. Clinical recognition and diagnosis of stiff person spectrum disorder are important early, as immunologic treatment options showing reliable efficacy in slowing disease progression are available. This case presents a wide constellation of symptoms in which we describe the pathophysiology, clinical diagnosis, and current as well as future treatment options that may be available.

## Introduction

Stiff person syndrome (SPS), previously referred to as stiff man syndrome, is a rare neuroimmunological autoimmune disorder that leads to gamma-aminobutyric acid (GABA) neuronal degeneration, resulting in stiffness, ataxia, and painful muscle spasms that are typically isolated to the proximal extremities [1]. This etiology was first described at the Mayo Clinic in 1956 in a report describing 14 patients over a 30-year period, all presenting with some form of stiffness, ataxia, and neurologic abnormalities that could not fit into another “acceptable” diagnosis [2]. Over the last 60 years, the syndrome has been better described to include disease progression involving pure cerebellar ataxia; progressive encephalomyelitis (PERM) causing hyperekplexia, myoclonus, brainstem, sensory, and/or autonomic dysfunction; and other autoimmune conditions [1,3].

The involvement of several other pathologies has led to the nomenclature of stiff person spectrum disorder (SPSD), in which there is heterogeneity between the conditions with the presence of glutamic acid decarboxylase 65-kilodalton isoform (GAD65) autoantibodies [1]. GAD65, the rate-limiting enzyme for GABA synthesis, has been well described in relation to autoimmune conditions, including, but not limited to, diabetes mellitus type-1 (DM-1), pernicious anemia (PA), autoimmune thyroiditis, seizure disorders, and SPS [4-7]. Therefore, it may be feasible for a syndromic person to be affected by multiple disease processes, as higher titers of GAD65 (>20 nmol/L) are more likely to cause multiple organ involvement [8]. Alternatively, high-titer patients are assessed based on presenting symptoms, neurological and/or non-neurological [8].

## Case presentation

A 29-year-old wheelchair-using female with a BMI of 37 presented to our clinic with a history of PA, partial complex seizures, SPS, DM-1, chronic hypothyroidism, anxiety, depression, and menorrhagia. The patient was initially diagnosed with PA at 17 years old, which was treated accordingly with cyanocobalamin, leading to the resolution of her symptoms of exhaustion, shortness of breath, difficulty concentrating, and mild numbness in the tips of her toes and fingers. Over the next two years, she began to have frequent, seizure-like episodes complicated by oxygen desaturation, pain and stiffness in her legs, ataxia leading to frequent falls, ophthalmoplegia, and blindness. She began experiencing episodes of “board-like” stiffness in her ankles, which did not allow for active or passive movement of the joint, as well as severe stiffness in both knees, resulting in confinement to a wheelchair. These episodes prompted testing for paraneoplastic syndromes, including antinuclear antibody (ANA), proteinase 3 antineutrophil cytoplasmic antibody (ANCA), myeloperoxidase ANCA, anti-double-stranded DNA (dsDNA), rheumatoid factor (RF), and anti-cyclic citrullinated peptide (CCP); all of which were negative. This led to testing for GAD65 autoantibodies, with positive results in both cerebrospinal fluid and serum samples across multiple tests, reaching levels as high as 2141 nmol/L.  At 19 years old, the patient was diagnosed with SPS based on the criteria presented in the discussion [1]. Subsequently, treatment to combat the disease progression of SPS was started. The patient received 2 g/kg of intravenous immunoglobulin (IVIG) every two weeks. However, the infusions were poorly tolerated due to severe debilitating headaches immediately after the infusions began. As a result of these headaches, rituximab infusions were trialed, with a baseline dosage of 1000 mg. This was later aborted due to severe infusion reactions, including headaches and urticaria. Intravenous methylprednisolone was added to the regimen; however, this was discontinued after discussion with the patient, as she believed no benefit was evident in reducing her stiffness, spasms of the lower extremities, or ability to ambulate. Eventually, the benefits of IVIG therapy were seen to overshadow the infusion-induced headaches, and IVIG therapy became the primary treatment. There was a noticeable response to IVIG, reducing stiffness in her knees and ankles, as well as an improved ability to utilize her upper extremities and turn her head from side to side; all of which were not functional prior to starting IVIG therapy. Symptomatic treatment of seizure-like episodes has been well-controlled with levetiracetam, although an initial electroencephalogram failed to display focal or epileptiform abnormalities. Her anxiety and depression symptoms continued to be controlled with diazepam and clonazepam.

Her condition was complicated by multiple prolonged hospital stays. At 29 years old, a tracheostomy with percutaneous endoscopic gastrostomy (PEG) tube placement was performed due to prolonged airway compromise, ventilator dependence, and seizure-like activities that led to frequent oxygen desaturation complicated by an episode of hospital-acquired pneumonia. The tracheostomy and PEG tube were reversed several months later due to frequent irritation, episodic hypoxemia, and increased anxiety.

In addition to her SPS diagnosis, careful management has been necessary to address other complications stemming from GAD65 autoimmunity. The patient has required close attention to her chronic hyperglycemia as a result of DM-1, with a hemoglobin A1C ranging from 6.2 to 6.6%. To manage this, she has required up-titration from 60 to 100 IU of insulin daily, which has been complicated by episodes of severe hypoglycemia, for which she has been educated on the utilization of juice and/or glucagon if necessary. Her PA has been well controlled with cyanocobalamin dosed to 1,000 mcg/mL weekly. Hypothyroidism was diagnosed at 23 years old and has been treated with 50 mcg tablets daily of levothyroxine, which has provided adequate control. Episodic seizures and anxiety have been adequately controlled with 80 mg of diazepam daily, supplemented by 6 mg of clonazepam. We are currently working on down-titrating diazepam, which has recently achieved a reduction to 60 mg daily.   Recently, the patient has been receiving blood transfusions for hemoglobin levels that are chronically below 7.0 g/dL, ranging from 4.4 to 6.7 g/dL. The chronic anemia has been attributed to a combination of PA and menorrhagia, which may be associated with a gynecologic condition. She is currently taking 150 mg/mL of medroxyprogesterone, which has failed to improve the severity of blood loss during menstruation.

Recent visits with a new neurologist led to questioning if SPS was correctly diagnosed in this patient. This led to a prolonged discussion that the current presentation of seemingly absent weakness and ataxia is due to treatment with IVIG, benzodiazepines, and opiates leading to alleviation of symptoms. The current plan is to wean the patient off IVIG and benzodiazepines to see if symptoms of SPS return more clearly.  The goal of achieving this would be to clarify whether the medical management was resolving SPS or whether the symptomology was due to another underlying etiology per discussion with her new neurologist.

## Discussion

SPS is based on a clinical diagnosis of (a) classical features, including muscular rigidity, EMG confirmation of co-contraction of agonist and antagonist muscles, episodic unexpected spasms, and absence of other neurologic disease that could explain this etiology; (b) supportive features, including anti-GAD65 antibodies, clinical response to benzodiazepines, and other endocrinopathies [1]. This is not a unified diagnosis but rather one that is seen across literature [1,4,5,7].

The pathophysiology of GAD65 autoimmunity is complex, with an association of a plethora of comorbidities. GABA is the universal inhibitory neurotransmitter of the central nervous system and relies on GAD65 for its production [5]. When there is a reduction of GABA, there is reduced control over the stimulation of the central nervous system, which can lead to overactivity of motor neurons, and decreased control over basal ganglia, resulting in poor control of direct and indirect pathways and psychiatric processes [4]. GAD65 is an enzyme that is present in many cell types, with high association with pancreatic beta-cells, thyroid follicular cells, and gastric parietal cells [6]. Collectively, this is why the syndrome is commonly observed with DM-1, PA, PERM, psychiatric abnormalities, stiffness, ataxia, and ocular detriment in SPSD.

The case described above began as isolated PA. PA is commonly associated with SPSD, with the highest association second to DM-1 [5]. While the mechanism is incompletely understood, the association is likely due to the antigen-presenting cells recognizing the anti-GAD65 antibodies. These antibodies are associated with GAD65 within parietal cells. As a result, this chronic recognition may lead to the development of antibodies against H+/K+-ATPase and intrinsic factor intracellularly during protein transport, leading to a reduction in the expression of these proteins from parietal cells [9,10]. 

Later, our patient developed DM-1, further contributing to her syndrome. DM-1 may be the most associated autoimmune condition within SPSD, occurring in nearly 80% of patients with SPS [7]. As explained above, this may be associated with the miseducation of antigen-presenting cells that leads to incorrect recognition of pancreatic beta cell components, resulting in an immune response and destroying the beta cells [9,10]. When associated with SPSD, GAD65 antibodies are greater than 1000 IU/mL or 20 nmol/L, which is 100-fold higher than observed in DM-1 as a standalone etiology [11]. This can aid in the diagnosis of SPSD in patients presenting with neurologic deficits, as well as hyperglycemia due to DM-1. 

Excessive deficiency of GABA also plays a large role in cerebral function that can lead to seizure activity, oculomotor abnormalities, visual deficits, as well as psychiatric complications [2]. Psychiatric complications, particularly anxious behavior, have been reported to trigger episodes of stiffness through heightened muscular activation, resulting in increased frequency of such episodes [12]. Autoimmune epilepsy alongside extreme anxiety may increase seizure activity, which is well documented in association with GAD65 antibodies [2,11,13]. These antibodies have been highly associated with damage to hippocampal regions that may result in mesial temporal sclerosis [11,13]. The current treatment protocol can be seen in Table 1; although this is not a universally accepted protocol, it is understandable that treatment is confined to GABAergic drugs and immunotherapy.

**Table 1:** Variable treatment options for stiff person syndrome *If benefit from IVIG, then maintenance with 1-2 g/kg every 4-6 weeks **If benefit, repeat every 6-12 months

Treatment Options	Mechanism of Action	Implication in Disease Process
1. Benzodiazepines	-	-
Diazepam	Long-Acting GABA-A Receptor Agonist	Reducing stiffness
Clonazepam	Intermediate-Acting GABA-A Receptor Agonist	Controlling seizure-like activity and anxiety
2. Central Acting Spasmodic	-	-
Baclofen	GABA-B Receptor Agonist	Reducing muscle stiffness and spasms
Tizanidine	Alpha-2 Receptor Agonist	Reducing muscle stiffness and spasms
3. Anti-Epileptics	-	-
Gabapentin/Pregabalin	Presynaptic antagonist of voltage-gated Ca2+ channels	Reduces neuropathic pain, spasms, seizure-like activity
Levetiracetam	Synaptic vesicle protein 2A (SV2A) agonist – reducing glutamatergic transmission	Reduces the occurrence of seizure-like activity
4. Immunotherapies	-	-
IVIG* (2 g/kg/month)	Binding Fc Receptor of GAD65 autoantibodies	Slows/reverses processes targeted by GAD65 autoantibodies including stiffness and spasms
Rituximab** (2 g over 15 days)	Antagonist of Cluster of Differentiation 20 (CD20) Receptor on B Lymphocytes – Reducing Antibody Production	Slows disease progression associated with GAD65 including stiffness and spasms
5. Other	-	-
Plasmapheresis	Filters GAD65 Autoantibodies Out of Plasma	Reduces severity of acute exacerbations of stiffness and spasms

In reviewing recent literature from January 1, 2020, through December 31, 2024, using PubMed with the keywords "Stiff Person Syndrome," "treatment," "benzodiazepine," "pharmacotherapy," and "immunotherapy," a total of 166 articles were identified. After a preliminary screening, 19 articles were deemed applicable, and further refinement based on therapeutic interventions related to first-line treatments for SPS led to a final selection of 9 studies (Table 2). These studies included systematic reviews, case reports, and comprehensive reviews, providing valuable insights into the management of SPS.

**Table 2:** A literature review of first-line treatment options for stiff person syndrome using case reports, systematic reviews, and literature reviews Abbreviations: IVIG: Intravenous immunoglobulin, Full responder (FR), Partial responder (PR), and Non-responder (NR)

Article	Benzodiazepine	IVIG
1. Dalakas MC, Yi J. [12]	Diazepam: 10 mg TID, 5 mg BID, 2.5 mg nightly, 5 mg daily. Clonazepam: 1 mg QID, 0.5 mg nightly, 0.5 mg BID.	Standard IVIG courses administered (exact doses not provided).
a. Efficacy	Effective but some experienced somnolence. Some tolerance.	FR: 3, PR: 3, NR: 2
b. Length	Ongoing with adjustments.	Varies; maintenance therapy based on response.
2. Yi J, Dalakas MC. [14]	Clonazepam: 0.5-2 mg as needed	2 g/kg over 2-5 days, maintenance every 4-6 weeks.
a. Efficacy	Effective for symptom management.	Significant improvement in muscle stiffness; some required long-term therapy.
b. Length	Ongoing	Ongoing
3. Newsome SD, Johnson T. [2]	Benzodiazepines used primarily for symptomatic relief at variable doses	Standard IVIG courses administered (2 g/kg)
a. Efficacy	Varies; may not address the underlying condition.	IVIG showed various effectiveness in symptom relief.
b. Length	Ongoing	Ongoing
4. Dalakas MC. [3]	Clonazepam/Diazepam: Commonly used, tailored dosing based on tolerance.	2 g/kg for 2-5 days
a. Efficacy	Generally effective; somnolence and tolerance issues noted.	Patients showed varying responses; some required ongoing therapy.
5. Yadav R et al. [15]	Benzodiazepines used primarily for symptomatic relief at variable doses	2 g/kg IVIG for several days; maintenance doses based on response.
a. Efficacy	Limited by side effects; ongoing monitoring required.	Improved symptoms but variable responses; some patients maintained long-term therapy.
6. Bose S et al. [16]	Clonazepam: Commonly used; effective for many patients.	Standard IVIG courses administered (exact doses not provided).
a. Efficacy	Some patients benefited while others experienced side effects.	A positive response was noted; some patients required adjustments over time.
7. Tsiortou P et al. [17]	Benzodiazepines used primarily for symptomatic relief at variable doses	Standard IVIG courses administered (exact doses not provided).
a. Efficacy	Some patients benefited while others experienced side effects.	IVIG was shown to significantly aid in managing symptoms; responses varied widely indicating a need for a more tailored approach.
8. Ortiz JF et al. [18]	Benzodiazepines used primarily for symptomatic relief at variable doses	Standard IVIG courses administered (exact doses not provided).
a. Efficacy	Often helps with symptoms but is limited by side effects. Effectiveness was variable.	Generally positive; ongoing adjustments may be necessary based on patient response
9. Vlad B et al. [13]	Benzodiazepines used primarily for symptomatic relief at variable doses	Standard IVIG courses administered (exact doses not provided)
a. Efficacy	Variable; patient specific.	IVIG remains a primary treatment option; responses are individualized and variable.

Intravenous immunoglobulin (IVIG) is increasingly recognized as a first-line treatment for SPS, with many studies reporting significant improvements in symptoms. The literature reveals that IVIG is generally administered in doses of 2 g/kg, either as a one-time treatment or in 4-6-week maintenance regimens based on individual patient responses (Table 2) [1,4,14-20]. While some patients respond well and maintain long-term benefits, others experience variable responses, highlighting the necessity for personalized treatment plans. One study focusing on IVIG treatment found that early initiation of IVIG therapy correlated with better long-term outcomes. In contrast, delayed treatment or waiting for more severe symptoms to develop resulted in a poorer response to IVIG, with little ability to reverse any established symptoms [14]. A clinical decline after years of treatment therapy was also observed in certain individuals, requiring a reassessment of therapeutic strategies. Collectively, these studies underscore the importance of tailored therapeutic strategies to optimize outcomes for patients with SPS being treated with IVIG.

Benzodiazepines, particularly clonazepam and diazepam, remain commonly used for symptomatic relief in SPS, with no definitive dosing regimen [2,4,14-20]. While these medications can effectively reduce muscle stiffness, spasms, and anxiety, their long-term efficacy is often limited by tolerance and side effects, especially in older patients and those at risk of falling. The need for ongoing monitoring and dosage adjustments, titrating to therapeutic response, and patient tolerance is emphasized, as excessive somnolence and tolerance issues frequently arise.

Future treatment has been aimed toward hematopoietic stem cell transplants (HSCT), as hematopoiesis is commonly disrupted in GAD65 autoimmunity [21]. In comparison to other immunotherapies, the addition of HSCT yielded variable results in their study populations. However, this may be an option moving forward for patients who are non-responsive to other immunotherapies [21,22].

## Conclusions

SPSD is a disease etiology that is rarely encountered in a clinical setting, and even more rarely described. As a spectrum, the disease has evolved to include several GAD65 autoantibody conditions, including DM1, PA, stiffness, and encephalopathy. We present a case that presents several of these phenomena that are currently responding to high-dose benzodiazepines and IVIG therapy. This reinforces current treatment guidelines. However, it is important to be aware of the spectrum that may be clinically present when making this diagnosis, which may alter the current treatment options. Furthermore, it is worth exploring further treatment options that may aid in blunting the progression of the disease and even the potential reversal of some of the pathologies present. We propose this paper for the purpose of igniting conversation about the diversity of presentation in patients with SPSD, as well as how to most appropriately manage these patients.
